# MC1R is dispensable for the proteinuria reducing and glomerular protective effect of melanocortin therapy

**DOI:** 10.1038/srep27589

**Published:** 2016-06-08

**Authors:** Yingjin Qiao, Anna-Lena Berg, Pei Wang, Yan Ge, Songxia Quan, Sijie Zhou, Hai Wang, Zhangsuo Liu, Rujun Gong

**Affiliations:** 1 Institute of Nephrology, Blood Purification Center, the First Affiliated Hospital of Zhengzhou University, Zhengzhou, China; 2 Division of Kidney Disease and Hypertension, Department of Medicine, Rhode Island Hospital, Brown University School of Medicine, Providence, Rhode Island, USA; 3 Department of Nephrology, Lund University Hospital, Lund, Sweden; 4 Department of Pathology, Rhode Island Hospital, Brown University School of Medicine, Providence, Rhode Island, USA

## Abstract

Melanocortin therapy by using adrenocorticotropic hormone (ACTH) or non-steroidogenic melanocortin peptides attenuates proteinuria and glomerular injury in experimental glomerular diseases and induces remission of nephrotic syndrome in patients with diverse glomerulopathies, even those resistant to steroids. The underlying mechanism remains elusive, but the role of melanocortin 1 receptor (MC1R) has been implicated and was examined here. Four patients with congenital red hair color and nephrotic syndrome caused by idiopathic membranous nephropathy or focal segmental glomerulosclerosis were confirmed by gene sequencing to bear dominant-negative MC1R mutations. Despite prior corticosteroid resistance, all patients responded to ACTH monotherapy and ultimately achieved clinical remission, inferring a steroidogenic-independent and MC1R-dispensable anti-proteinuric effect of melanocortin signaling. In confirmatory animal studies, the protective effect of [Nle^4^, D-Phe^7^]-α-melanocyte stimulating hormone (NDP-MSH), a potent non-steroidogenic pan-melanocortin receptor agonist, on the lipopolysaccharide elicited podocytopathy was completely preserved in MC1R-null mice, marked by reduced albuminuria and diminished histologic signs of podocyte injury. Moreover, in complementary *in vitro* studies, NDP-MSH attenuated the lipopolysaccharide elicited apoptosis, hypermotility and impairment of filtration barrier function equally in primary podocytes derived from MC1R-null and wild-type mice. Collectively, our findings suggest that melanocortin therapy confers a proteinuria reducing and podoprotective effect in proteinuric glomerulopathies *via* MC1R-independent mechanisms.

Proteinuria, the hallmark of glomerular injury, is a common finding on urinalysis, and is by itself a strong, independent and modifiable risk factor for end stage renal disease, premature death of cardiovascular origin, and ischemic stroke in patients with diabetes^1^,^2^,^3^,^4^. Despite recent advances in angiotensin blockade and immunosuppression^5^,^6^,^7^, refractory proteinuria continues to be a challenge in clinical practice. It is imperative to develop more effective modalities to ameliorate glomerular injury and induce remission of proteinuria. Recently, a plethora of evidence points to melanocortin system as a novel target for treatment of proteinuria^8^.

The melanocortin system is a set of neuropeptidergic and immunoendocrine signaling pathways that play an integral role in the homeostatic control of a diverse array of physiological functions, including melanogenesis, stress response, inflammation, immunomodulation, adrenocortical steroidogenesis and more^8^. It consists of multiple components, including the five G protein-coupled melanocortin receptors: melanocortin receptor 1 (MC1R) to MC5R; peptide ligands: α, β, γ- melanocyte stimulating hormone (α, β, γ- MSH), adrenocorticotropic hormone (ACTH) secreted by the anterior pituitary; and endogenous antagonists^9^,^10^,^11^. The biological functions of melanocortin system are mediated by the five melanocortin receptors (MCRs), which have distinct tissue distribution, convey different signaling and exert varying biological activities in different organ systems^10^,^11^,^12^,^13^. Steroidogenesis, the most well-known melanocortin function, is triggered only by ACTH and mediated *via* MC2R in the adrenal cortex^10^,^12^

As one of the 4 native melanocotin peptides, ACTH has been widely used since the 1950s for the treatment of nephrotic syndrome^14^,^15^, but was later replaced by synthetic corticosteroids, because steroids were inexpensive and easier for administration. However, recent clinical data indicate that ACTH is still effective in patients with steroid resistant nephrotic syndrome^16^,^17^,^18^,^19^,^20^, suggestive of a steroidogenic-independent melanocortin mechanism. Moreover, the anti-proteinuric effect of ACTH seems not limited to a particular type of glomerular disease, but observed in diverse glomerulopathies, including membranous nephropathy (MN)^16^,^18^,^19^,^21^,^22^, minimal change disease (MCD)^18^,^19^,^21^, focal segmental glomerulosclerosis (FSGS)^18^,^19^,^20^,^21^,^23^ and IgA nephropathy^19^, implying that the melanocortin effect might, at least in part, target a pathogenic pathway common to all proteinuric kidney diseases. Of note, podocyte, as a critical component of the glomerular filtration barrier controlling glomerular permselectivity, is a major culprit accounting for massive proteinuria in diverse glomerular diseases^24^,^25^,^26^,^27^. Converging evidence suggests that the beneficial effect of melanocortin therapy is likely attributable to a direct action on podocytes^28^,^29^. Indeed, a recent study demonstrated that MC1R is predominantly expressed in glomerular podocytes in rodents and humans^28^. By using synthetic MC1R agonists, it was suggested that MC1R agonism reduces proteinuria and improves glomerular morphology^28^. However, this study conflicts with data from our and other groups indicating that other MCR, rather than MC1R, is dominant in glomeruli and kidneys^30^,^31^,^32^,^33^. Furthermore, this study is not conclusive because the conclusion exclusively relied on the synthetic MC1R agonist, which actually provides poor discrimination between different types of MCR apart from potential unknown properties^34^. Therefore, more conclusive evidence, such as the use of mutants with selective MCR deficiency, is essential to define and validate the role of MC1R in mediating the beneficial effect of melanocortin therapy in glomerular disease.

MC1R, abundantly expressed by melanocytes in the skin^35^, is a key control point in melanogenesis and determines hair color^36^. Loss-of-function or null mutations in MC1R are associated with a switch from eumelanin to phaeomelanin production, resulting in a red or yellow coat color^37^,^38^,^39^. Mice with the null mutation in MC1R, also known as the recessive yellow mice, are homozygous for the MC1R^e^ allele and are phenotypically normal under physiologic conditions^37^,^38^. Akin to yellow fur in MC1R^e/e^ mice, humans with MC1R mutations have red hair color (RHC)^39^ (prevalence: 10% to 30%^40^,^41^) but are otherwise normal. This study harnessed the naturally occurring MC1R mutations in both humans and mice to define the role of MC1R in mediating the effect of melanocortin therapy in proteinuric glomerular disease.

## Results

### Congenital RHC in humans is associated with dominant-negative mutations in MC1R

**Figure 1:**
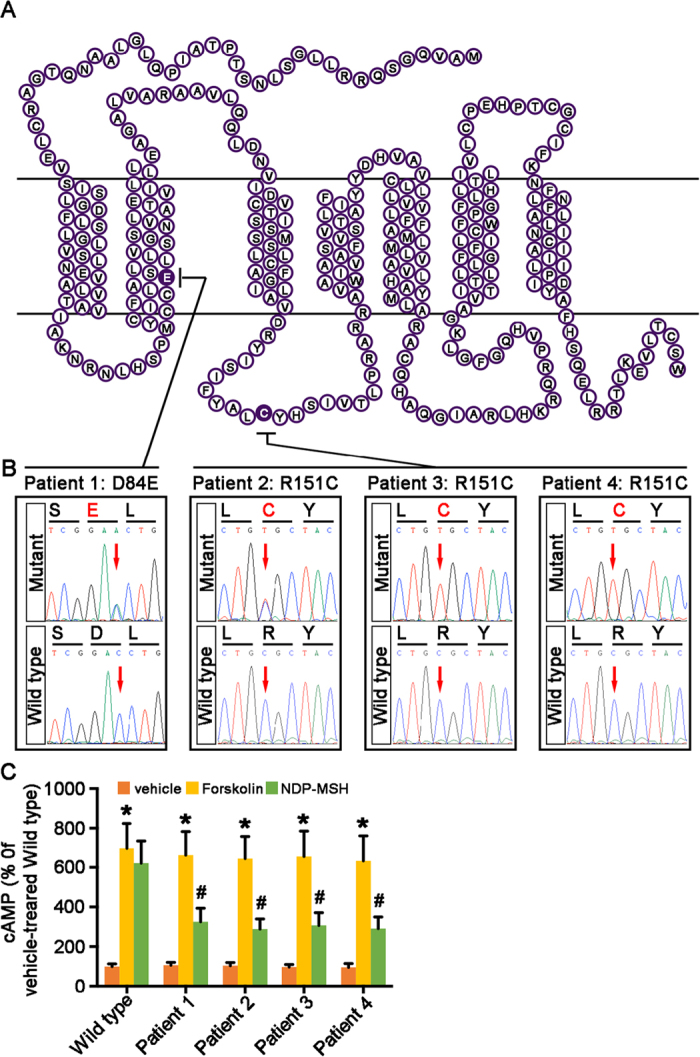
Nephrotic patients with congenital red hair color carry dominant-negative mutations in MC1R. Blood specimens from 4 red-haired nephrotic patients were collected and processed for Sanger DNA sequencing of the protein-coding region of MC1R gene. (**A**) Schematic diagram of the protein structure of MC1R depicting the amino acid sequence. A single amino acid substitution deduced from MC1R gene sequence was found in the 4 patients respectively and was highlighted in black. A substitution of Aspartate (D) by Glutamate (E) at amino acid position 84 was found in patient 1, a substitution of Arginine (R) by Cysteine (C) at amino acid position 151 in patient 2, 3 and 4. (**B**) Partial sequencing chromatograms reveal heterozygous C252A point mutation in patient 1, heterozygous C451T mutation in patient 2, and homozygous C451T mutation in patients 3 and 4, all resulting in antimorphic dominant-negative MC1R alleles responsible for the phenotype of red hair color. (**C**) PBMCs, prepared from the 4 red-haired patients and a control individual with wild-type MC1R, were treated with forskolin (10 μM) or NDP-MSH (10^−7^ M), a potent non-steroidogenic pan-MCR agonist, for 6 hours followed by the cAMP assay. Vehicle-induced cAMP formation in PBMCs derived from the wild-type individual was defined as 100%. ^#^*P* < 0.05 *vs* NDP-MSH-treated wild-type PBMCs (n = 6); *P < 0.05 *vs* vehicle-treated PBMCs for each study subject (n = 6).

Totally 80 patients with nephrotic syndrome had been treated with ACTH at the renal clinics of Lund University Hospital in the past 6 years and were retrospectively screened. Four red-haired patients were identified (Table 1) to have congenital RHC, freckles, and fair skin (Fitzpatrick skin type 1). Blood specimens were prepared for Sanger sequencing to determine MC1R mutation status. Patient 1, a 65-year-old male, was found to carry C252A heterozygous point mutation, which resulted in the substitution of Aspartate (D) by Glutamate (E) at amino acid position 84 in the MC1R protein (D84E). Patient 2, a 45-year old female, carried C451T heterozygous point mutation, leading to the substitution of Arginine (R) by Cysteine (C) at amino acid position 151 in the MC1R protein (R151C). Patient 3, a male aged 18, and patient 4, a male aged 53, both carried C451T homozygous mutation, leading to the R151C variant of MC1R (Fig. 1). The D84E and R151C mutations have been known to exert dominant-negative effects on both expression and function of MC1R, incurring the null phenotype of MC1R^42^,^43^,^44^. To validate the functional deficiency of MC1R in these patients, peripheral blood mononuclear cells (PBMCs) known to express MC1R^45^,^46^,^47^ were prepared and stimulated with [Nle^4^, D-Phe^7^]-α-melanocyte stimulating hormone (NDP-MSH), a potent non-steroidogenic pan-MCR agonist^48^. Despite an intact cyclic adenosine monophosphate (cAMP) response to forskolin treatment in all PBMCs, the NDP-MSH triggered cAMP induction was prominently diminished in PBMCs prepared from the 4 red-haired patients as compared with that in PBMCs derived from a control individual with wild-type MC1R (Fig. 1C), suggesting an impairment of MC1R-mediated cAMP response in the patients.

### ACTH monotherapy induces remission of proteinuria in MC1R-mutant patients with steroid-resistant nephrotic syndrome

**Figure 2:**
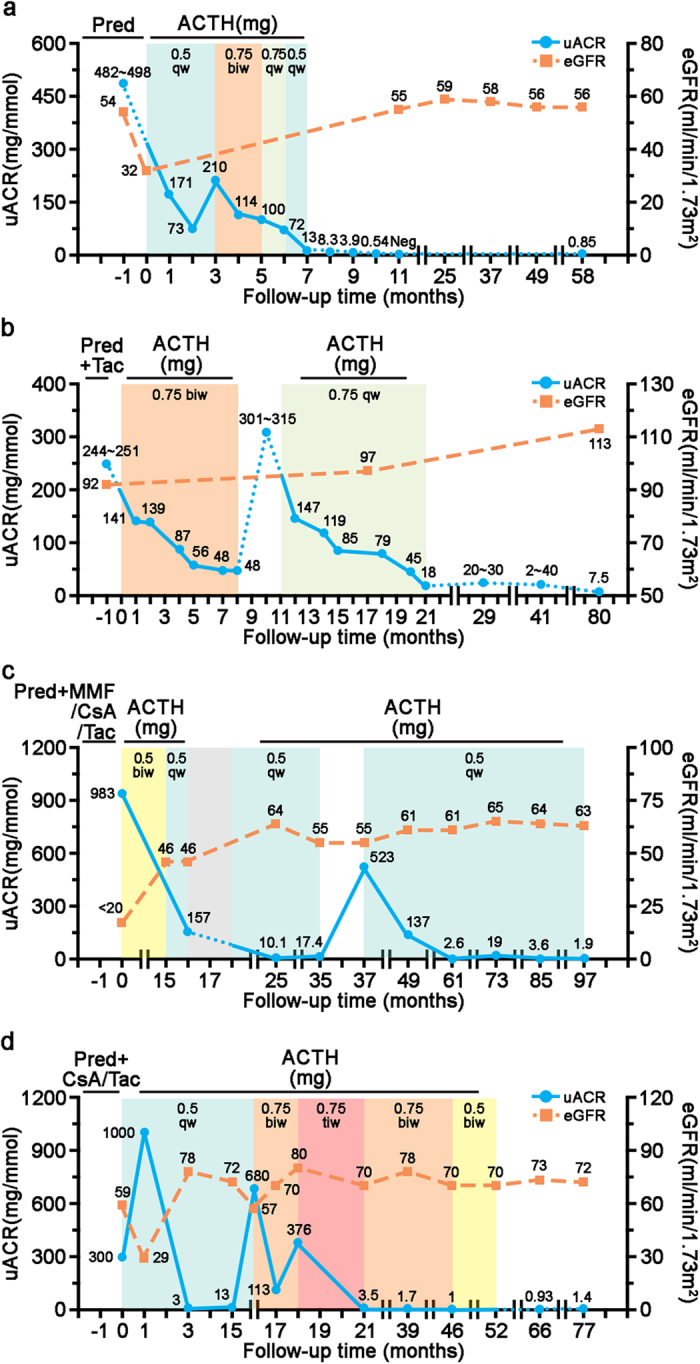
Trend of proteinuria and renal function in red-haired nephrotic patients upon ACTH monotherapy. (**a**) Patient 1 with iMN had been refractory to prednisolone (Pred). ACTH was given at an initial dose of 0.5 mg once a week (qw) with progressive reduction in proteinuria. When a rebound of proteinuria was noted, the dose of ACTH was increased to 0.75 mg twice a week (biw) for 2 months, paralleled by further improvement of nephrotic syndrome. The patient was maintained on ACTH for another 2 months at a reduced dose (0.5 mg once a week), and a complete remission was achieved. He was followed up for more than 4 years with sustained remission and stable kidney function as shown by estimated glomerular filtration rate (eGFR). (**b**) Patient 2, diagnosed with iMN, was resistant to prednisolone plus tacrolimus (Tac). ACTH monotherapy was initiated with progressive improvement in proteinuria. A rapid relapse of nephrotic syndrome ensued when ACTH treatment was discontinued 8 months later. Subsequently, ACTH therapy was reinstated at a lower dose until complete remission was achieved. She was followed up for more than 5 years without relapse. (**c**) Patient 3, diagnosed with FSGS, was changed to ACTH monotherapy after suspected calcineurin nephrotoxicity. A dose reduction after ACTH therapy for 3 years resulted in a severe relapse. To date, this patient has maintained complete remission with a low-dose ACTH. ACTH treatment was paused for 2 month (grey area) due to pulmonary infection. (**d**) Patient 4, diagnosed with FSGS, remitted quickly in 3 months after initiation of ACTH. The dose was later escalated two times upon two episodes of relapse. ACTH was tapered off slowly over a period of 31 months. After stopping ACTH, this patient was followed up for over 2 years without relapse. All patients were simultaneously treated with ACEI or ARB and they tolerated ACTH therapy very well, except that patient 3 experienced one episode of fungal lung infection (Fig. 2c, grey area). MMF, mycophenolate mofetil; CsA, cyclosporine A.

Patient 1 and 2 were diagnosed with idiopathic membranous nephropathy (iMN), and patient 3 and 4 primary FSGS, as proven by kidney biopsy. All patients had been treated with immunosuppressive regimens comprising prednisolone, cyclosporine A, tacrolimus or mycophenolate mofetil (MMF), in addition to angiotensin-converting enzyme inhibitors (ACEI) or angiotensin II receptor blockers (ARB). After having been on immunosuppressants in adequate dosages for a sufficient length of time, patients exhibited refractory response in proteinuria or nephrotic syndrome and manifested with severe adverse effects (Table 1). Thereafter, the 4 patients discontinued all immunosuppressants and were converted to synthetic ACTH monotherapy (Synacthen Depot, Swedish Orphan Biovitrum AB, Stockholm, Sweden) in the presence of ACEI or ARB treatment. Following ACTH therapy, patient 1 demonstrated a progressive reduction in proteinuria, paralleled by an improved kidney function measured by the estimated glomerular filtration rate (eGFR) as calculated using the CKD-EPI [CKD Epidemiology Collaboration] creatinine equation^49^. After 7 months of ACTH treatment, including dose escalation and de-escalation, this patient achieved complete remission of proteinuria, subsequently ceased therapy and was followed up for over 50 months without relapse of proteinuria (Fig. 2a). Patient 2 also experienced a quick and marked improvement in proteinuria following ACTH therapy, and attained complete remission at the end of 8 months. Afterwards, ACTH treatment was terminated, but a rapid relapse of proteinuria and nephotic syndrome ensued, suggesting that the initial remission was likely attributable to a therapeutic effect of ACTH rather than a spontaneous remission of iMN. ACTH therapy was subsequently reinstated at a lower dose, again leading to a persistent reduction of proteinuria, concomitant with a steadily improved kidney function. This patient achieved complete remission of proteinuria at the end of 21 months, stopped the medications and was followed up for 59 months with no signs of relapse (Fig. 2b). Patient 3 had a long history of nephrotic syndrome due to FSGS for over 16 years since childhood. He responded to ACTH monotherapy slowly but steadily with a sustained improvement in kidney function (Fig. 2c). The patient seems to be ACTH-dependent because a dose reduction 3 years after ACTH therapy resulted in a severe relapse. So far, this patient maintains complete remission with a low-dose ACTH. Patient 4 remitted quickly in 3 months after initiation of ACTH monotherapy. The dose of ACTH was escalated two times upon two episodes of relapse. After a sustained remission was achieved, ACTH was tapered off slowly over a period of 31 months (Fig. 2d). This patient was followed up for over 2 years after weaning off ACTH without relapse. All patients tolerated ACTH therapy very well, except that patient 3 experienced one episode of fungal lung infection (Fig. 2c, grey area). Taken together, despite the descriptive nature of the findings from these 4 patients, it seems that steroidogenesis is unlikely responsible for the beneficial effect of ACTH because all patients were refractory to antecedent corticosteroid treatments. More importantly, the MC1R dominant-negative mutations carried by these patients ruled out the contribution of MC1R signaling to the proteinuria reducing effect.

### Mice with the loss-of-function mutation in MC1R are healthy with normal kidney function and histology

**Figure 3:**
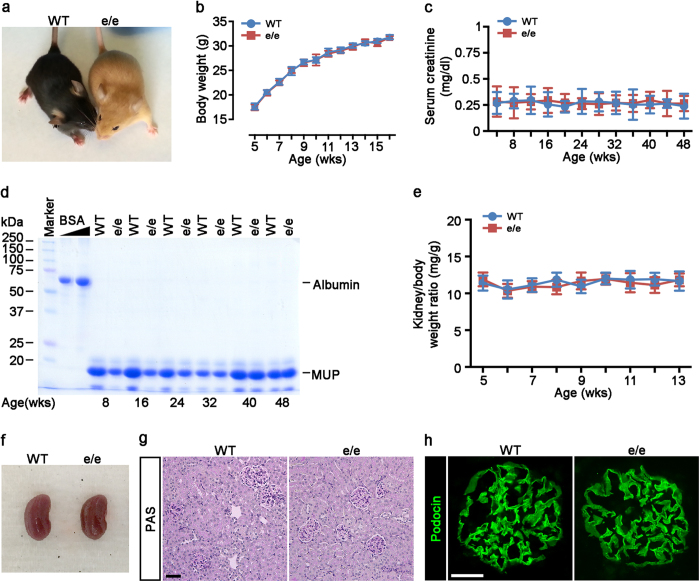
Mice with the loss-of-function mutation in MC1R are phenotypically normal with normal kidney function and histology. (**a**) Photo of an 8-week-old recessive yellow (MC1R^e/e^) mouse that bears the naturally occurring loss-of-function mutation in MC1R (on the right) together with a wild-type (WT) littermate (on the left) on the C57BL/6 genetic background. (b~c) Time-course changes in body weights (**b**), serum creatinine levels (**c**) in a representative litter of sex-matched (male) MC1R^e/e^ and WT mice. Not significant, MC1R^e/e^ versus WT (n = 6). (**d**) Urine was collected at the indicated ages from a representative litter of MC1R^e/e^ mice and WT littermates and urine samples (5 μl) were subjected to SDS-PAGE followed by Coomassie brilliant blue staining. Bovine serum albumin (BSA, 3 μg and 6 μg) served as standard control. MUP, mouse major urinary protein. (**e**) Time-course changes in Kidney to body weight ratios in a representative litter of sex-matched (male) MC1R^e/e^ and WT mice. Not significant, MC1R^e/e^ versus WT (n = 6). (**f**) Photo of kidneys excised from 8-week-old male MC1R^e/e^ and WT mice showing normal and similar gross appearance, size, and color. (**g**) MC1R^e/e^ and WT mice have comparable renal and glomerular histology, shown by PAS staining for light microscopy, Scale bar = 50 μm, (h) Similar distribution and expression of podocyte slit diaphragm protein podocin in renal glomeruli in MC1R^e/e^ and WT mice shown by fluorescent immunohistochemistry staining. Scale bar = 20 μm.

**Figure 4:**
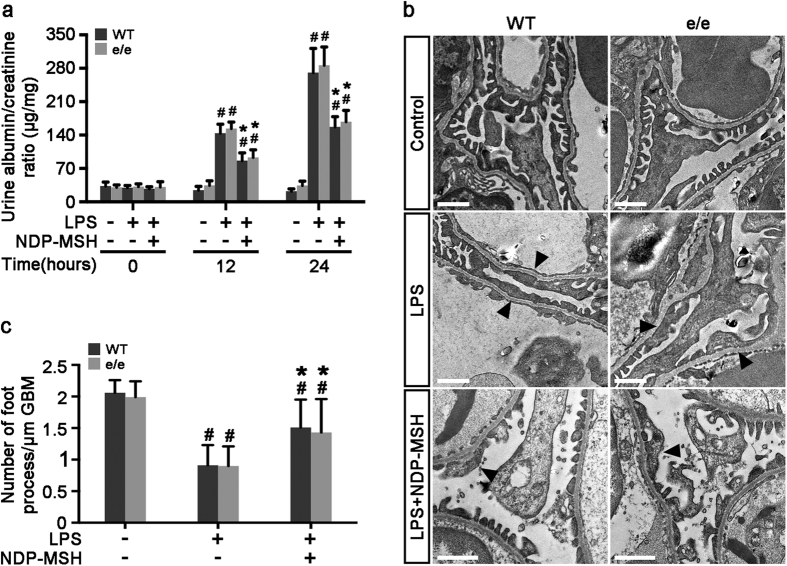
The protective effect of NDP-MSH on the LPS-elicited proteinuria and podocytopathy is completely preserved in mice null for MC1R. Male MC1R^e/e^ mice and wild-type (WT) littermates aged 8 weeks were treated with saline or lipopolysaccharide (LPS, 200 μg) intraperitoneally. One hour before and 12 h after LPS or saline injection, mice received subcutaneous injection of NDP-MSH (0.6  μmol/kg *wt*) or an equal amount of saline. Mice were followed for 24 h. (**a**) Urine was collected and processed for urine albumin ELISA assay adjusted for urine creatinine concentrations. ^#^*P* < 0.05 *vs* control group (n = 6); *P < 0.05 *vs* LPS group (n = 6). (**b**) Kidney cortical tissues were procured at 24 h and processed for transmission electron microscopy. LPS injury caused marked ultrastructural signs of podocytopathy, characterized by microvillous transformation and a variable degree of foot process effacement (black arrowheads). This effect was attenuated by NDP-MSH treatment equally in MC1R^e/e^ mice and WT littermates. Scale bar = 1 μm. (**c**) Morphometric analysis of the number of foot processes per micrometer of glomerular basement membrane (GBM) by electron microscopy. ^#^*P* < 0.01 *vs* control group (n = 6); *P < 0.05 *vs* LPS group (n = 6).

To further validate the MC1R-independent anti-proteinuric effect of melanocortin therapy, we harnessed a line of mice with the naturally occurring loss-of-function mutation in MC1R. Akin to RHC in humans with MC1R mutations, MC1R^e/e^ mice have yellow coat color instead of black coat color (Fig. 3a) in wild-type littermates (WT). Long period follow-up indicated that MC1R^e/e^ mice were viable and fertile with no noticeable difference from WT mice in gross appearance (Fig. 3a), behavior, development and body weight (Fig. 3b). Besides, kidney function, shown by serum creatinine levels (Fig. 3c) and urine albumin excretion (Fig. 3d), was normal in MC1R^e/e^ mice. In addition, both weight (Fig. 3e) and gross appearance (Fig. 3f) of the kidneys from MC1R^e/e^ mice were normal as compared with WT littermates. In consistency, kidney histology (Fig. 3g), expression of podocyte slit diaphragm protein podocin (Fig. 3h), and glomerular ultrastructure (Fig. 4) were all comparable between MC1R^e/e^ mice and control WT littermates.

### MC1R is not required for the proteinuria reducing and glomerular protective effect of NDP-MSH in lipopolysaccharide (LPS)-induced experimental podocytopathy

**Figure 5:**
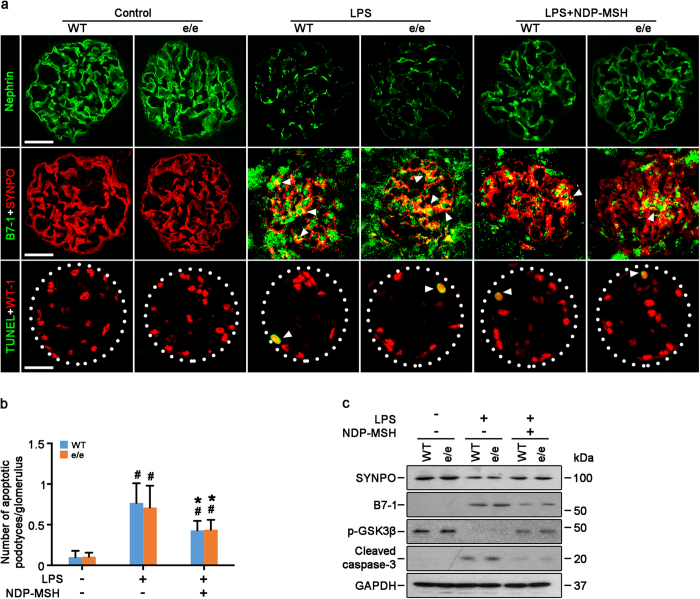
Molecular signs of podocytopathy in LPS-injured mice are ameliorated by NDP-MSH *via* an MC1R independent mechanism. MC1R^e/e^ mice and wild-type (WT) littermates were treated as stated in Fig. 4. (**a**) Kidney cortical tissues were procured at 24 h and processed for fluorescent immunohistochemistry staining for indicated molecules or TUNEL staining for apoptotic cells (white arrowheads). Scale bar = 20 μm. SYNPO, synaptopodin; (**b**) Absolute counting of the numbers of podocytes positive for both TUNEL and WT-1 as the means of 20 glomeruli. ^#^*P* < 0.01 *vs* control group (n = 6); *P < 0.05 *vs* LPS group (n = 6). (**c**) Glomeruli were isolated from the excised kidneys by the magnetic beads based approach and prepared for immunoblot analysis for indicated molecules.

To determine if MC1R plays a role in glomerular disease and in mediating the therapeutic effect of melanocortins, the murine model of LPS-induced albuminuria and podocyte injury was developed in WT and MC1R^e/e^ mice. LPS injury caused overt albuminuria at 24 h (Fig. 4a). This coincided with the ultrastructural lesions of glomerular podocytes on electron microscopy (Fig. 4b), characterized by microvillous transformation and a variable degree of foot process effacement, which was quantified by measuring the number of foot processes per unit length of glomerular basement membrane (Fig. 4c). Besides, fluorescent immunohistochemistry staining also demonstrated molecular evidence of podocyte injury and cytoskeleton disorganization in LPS injured kidneys, including loss of podocyte specific markers, like slit diaphragm protein nephrin and synaptopodin, an actin-associated protein essential for the integrity of actin cytoskeleton in podocytes. In addition, LPS-injured kidneys displayed podocyte *de novo* expression of the podocytopathic molecule B7-1, which functions at the interface between innate/adaptive immunity and podocyte cytoskeletal remodeling (Fig. 5a). Moreover, acute LPS injury also caused occasional cellular apoptosis in glomeruli, as shown by terminal deoxynucleotidyl transferase dUTP nick end labeling (TUNEL) staining, at the periphery of glomerular tufts in Wilms tumor 1 (WT-1)-positive cells, suggestive of podocyte apoptosis (Fig. 5a,b). The morphologic findings were further corroborated by immunoblot analysis of isolated glomeruli for synaptopodin, B7-1 and cleaved caspase-3 (Fig. 5c). Furthermore, inhibitory phosphorylation of glycogen synthase kinas (GSK) 3β, a kinase centrally involved in podocyte injury^50^, was prominently mitigated in isolated glomeruli, shown by immunoblot analysis (Fig. 5c). Following LPS injury, MC1R^e/e^ mice also developed albuminuria, podocyte ultrastructural lesions, and molecular signs of podocyte injury, to the same degree as observed in WT mice. NDP-MSH treatment attenuated the LPS elicited proteinuria by more than 40% in WT mice (Fig. 4a), concomitant with prominent amelioration of podocyte ultrastructural lesions (Fig. 4b,c), prevented loss of podocyte markers in glomeruli, diminished podocyte apoptosis and B7-1 expression, and restored inhibitory phosphorylation of GSK3β (Fig. 5). This beneficial effect of NDP-MSH was equally observed in LPS-injured MC1R^e/e^ mice with an improvement to the same extent in proteinuria, ultrastructural podocyte lesions (Fig. 4) and molecular signs of podocytopathy (Fig. 5). Altogether, regardless of the MC1R mutation status, mice developed podocytopathy upon LPS insult with equal severity, and melanocortin treatment attenuated proteinuria and ameliorated podocyte injury to the same extent.

### NDP-MSH protects against the LPS impaired filtration barrier function of podocyte monolayers *via* an MC1R independent mechanism

**Figure 6:**
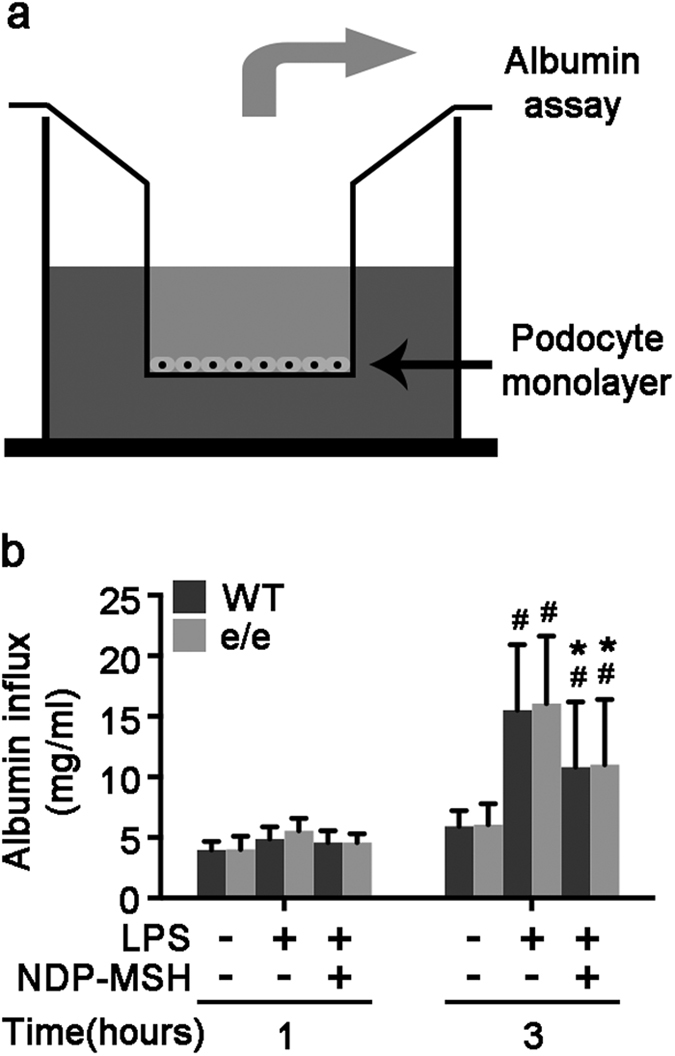
MC1R is not required for the protective effect of NDP-MSH on the LPS-impaired filtration barrier function of podocyte monolayers. Primary podocytes were prepared from glomeruli isolated from MC1R^e/e^ mice and wild-type (WT) littermates. Following different treatments, paracellular permeability assay was carried out to determine the filtration barrier function of podocytes monolayers. (**a**) A simplified schematic representation of the paracellular permeability assay adopted to assess the filtration barrier function of podocytes monolayers. Podocyte monolayers on collagen-coated Transwell filters were injured with LPS (20 μg/ml) or saline in the presence or absence of NDP-MSH (10^−7^ M) for 3 hours, and albumin permeability across podocyte monolayers was then determined. (**b**) Quantification of the albumin influx across podocyte monolayers. Duration of albumin incubation is shown on *x*-axis. ^#^*P* < 0.01 *vs* control group (n = 6); *P < 0.05 *vs* LPS group (n = 6).

To determine if the apparently MC1R-independent anti-proteinuric effect of melanocortin therapy stems, at least in part, from a direct effect on podocytes, *in vitro* studies on podocytes were next carried out. Primary podocytes were prepared from glomeruli isolated from WT and MC1R^e/e^ mice by the magnetic beads based approaches. A paracellular permeability influx assay was employed to assess the filtration barrier function of podocyte monolayers. Shown in Fig. 6a, this simple assay measured the albumin flux rate across the monolayers of podocytes cultured on collagen-coated Transwell filters. Compared with vehicle treatment, LPS injury induced a considerable albumin influx across the monolayers of podocytes derived from WT or MC1R^e/e^ mice to the same degree. This effect was partially abrogated by NDP-MSH to the same magnitude in podocytes derived from WT or MC1R^e/e^ mice (Fig. 6b), inferring that MC1R is unlikely required for the protective effect of melanocortin treatment on the filtration barrier function of podocytes.

### NDP-MSH promotes survival, preserves cytoskeleton integrity and mitigates hypermotility in podocytes in an MC1R-dispensable fashion

**Figure 7:**
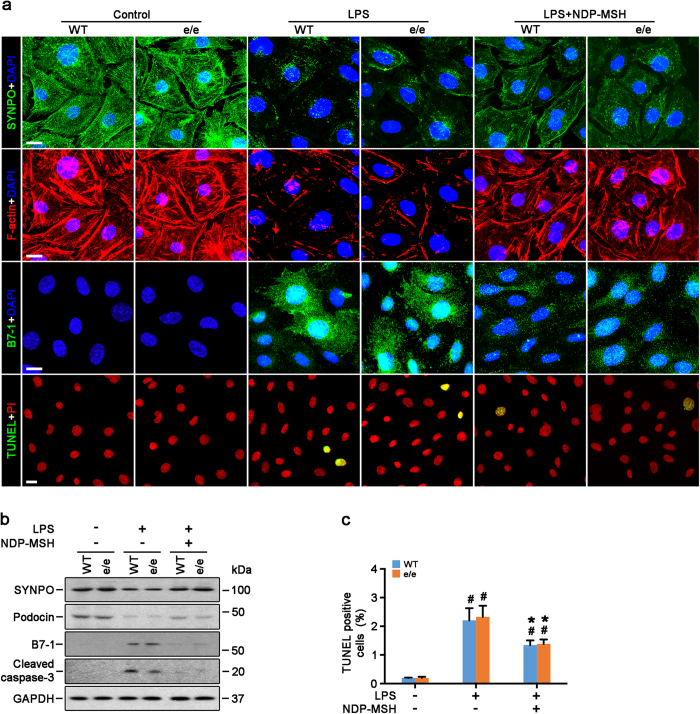
NDP-MSH promotes podocyte survival and preserves podocyte cytoskeleton integrity *via* an MC1R independent mechanism. Primary podocytes were prepared from glomeruli isolated from MC1R^e/e^ mice and wild-type (WT) littermates and injured with LPS (20 μg/ml) or saline in the presence or absence of NDP-MSH (10^−7^ M) for 24 hours. (**a**) Cells were fixed and subjected to fluorescent immunohistochemistry staining for indicated molecules or TUNEL staining for apoptotic cells. Scale bar = 20 μm; DAPI, 4′,6-diamidino-2-phenylindole; SYNPO, synaptopodin; PI, propidium iodide; (**b**) Cell lysates were prepared for immunoblot analysis for indicated molecules. (**c**) Absolute counting of the numbers of TUNEL positive apoptotic podocytes expressed as percentage of the total number of podocyte nuclei per high-power field. ^#^*P* < 0.05 *vs* control group (n = 6); *P < 0.05 *vs* LPS group (n = 6).

**Figure 8:**
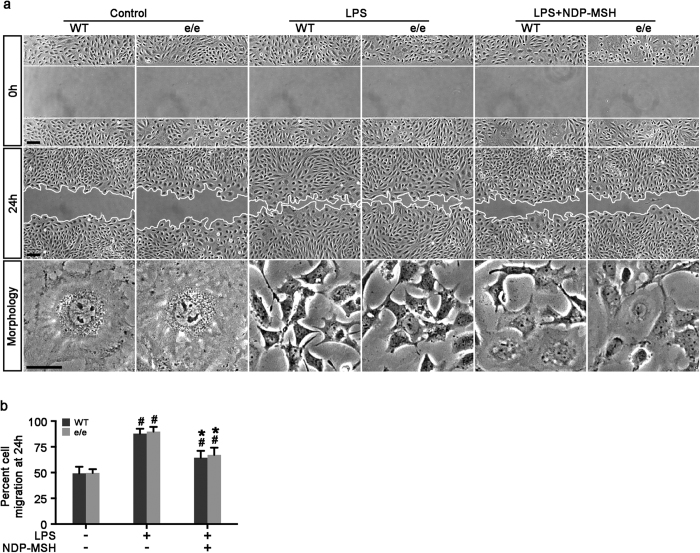
NDP-MSH attenuates podocyte hypermotility elicited by LPS to the same extent in primary podocytes derived from WT and MC1R null mice. Primary podocytes were prepared from glomeruli isolated from MC1R^e/e^ mice and WT littermates and injured with LPS (20 μg/ml) or saline in the presence or absence of NDP-MSH (10^−7^ M). Scratch wound was made immediately after LPS or saline treatment. (**a**) Phase-contrast micrographs were taken immediately after wounding (0 h) and after migration for 24 h. Scale bar = 100 μm. Cell morphology at 24 h was taken under high-power fields. LPS injury resulted in marked podocyte shrinkage and this effect was abrogated by NDP-MSH. Scale bar = 20 μm. (**b**) Quantification by computerized morphometric analysis of the cell migration area following the indicated treatments. ^#^*P* < 0.05 *vs* control group (n = 6); *P < 0.05 *vs* LPS group (n = 6).

Among the many cytopathic processes, impairment of cellular viability and cytoskeleton disorganization play key roles in podocyte dysfunction. As a prototype of pro-oxidants, LPS elicited apoptosis, as detected by TUNEL staining (Fig. 7a,c) and immunoblot analysis for cleaved caspase 3 (Fig. 7b), to the same extent in primary podocytes derived from WT or MC1R^e/e^ mice. This proapoptotic effect was mitigated by NDP-MSH treatment equally in podocytes derived from WT or MC1R^e/e^ mice (Fig. 7). Moreover, as shown by fluorescein phalloidin staining of filamentous actin (Fig. 7a), LPS elicited disruption of actin cytoskeleton integrity in primary podocytes that manifested as increased expression of cortical filaments and diminished ventral stress fibers. This coincided with reduced expression of podocin and the actin associated protein synaptopodin, and *de novo* expression of the podocytopathic molecule B7-1, shown by immunocytochemistry staining (Fig. 7a) or immunoblot analysis (Fig. 7b). The cytoskeleton-disruptive efficacy of LPS was observed to the same extent in primary podocytes derived from WT or MC1R^e/e^ mice and was equally attenuated by NDP-MSH treatment (Fig. 7a). Evidence suggests that podocyte cytoskeleton disarrangement affects podocyte motility and thereby causes podocyte dysfunction. To determine the functionality of the changes in podocyte cytoskeleton, a traditional cell migration assay for assessing cellular motility was employed (Fig. 8). Under basal conditions, podocytes possessed a basal migratory capacity that lessened the distances between the leading edges of the migrating podocyte sheets. LPS injury resulted in marked podocyte shrinkage and an asterlike cell shape, and strikingly accelerated closure of the gap between the invading fronts of the cells (Fig. 8a,b), denoting podocyte hypermotility. This effect was observed to the same degree in primary podocytes derived from WT or MC1R^e/e^ mice and was equally and partially attenuated by NDP-MSH treatment (Fig. 8).

## Discussion

A growing body of evidence recently suggests that the melanocortin based therapy is effective in reducing proteinuria in nephrotic glomerulopathies. The effectiveness of ACTH in patients with steroid resistant nephrotic syndrome, like the patients presented above, argues for a steroidogenic independent melanocortin mechanism responsible for proteinuria remission^16^,^17^,^18^,^19^,^20^,^21^,^22^,^23^. In agreement, non-steroidogenic melanocortin peptide α-MSH and its synthetic analogues conferred a potent anti-proteinuric effect in animal models of glomerular diseases^28^,^29^. Emerging data indicate that the anti-proteinuric effect of melanocortins is likely attributable, at least in part, to a direct protection of glomerular podocytes^28^,^29^. However, which type(s) of MCR is (are) responsible for the proteinuria reducing effect still remains a matter of debate. In this study, we demonstrated for the first time that MC1R is not essential for the proteinuria reducing and glomerular protective effect of melanocortin therapy. In agreement with our findings, no clinical evidence exists so far supporting any associations between RHC or genetic variations of MC1R and the susceptibility to nephrotic syndrome, proteinuria or glomerulopathies.

Our findings apparently conflict with those made by Lindskog *et al*.^28^, who, by using the synthetic MC1R agonist, suggested that MC1R mediates an anti-proteinuric effect. However, due to the high homology of the five MCR in both amino acid composition and molecular structure, some MCR agonists, even those claimed to be selective for a certain type of MCR, more or less have agonistic activities across other types of MCR^34^,^51^,^52^. For instance, MS05, the MC1R agonist used in the Lindskog study^28^, was actually able to activate MC3R in MC3R-expressing cells, resulting in a level of cAMP response comparable to that achieved by potent pan MCR agonists, like NDP-MSH or α-MSH^51^. Thus, the contribution of other MCR, like MC3R, to the proteinuria reducing effect cannot be ruled out. In this study, red-haired patients with nephrotic syndrome responded well to ACTH therapy, despite resistance to prior corticosteroids. All patients carried the MC1R dominant-negative mutations (D84E and R151C)^42^. The molecular mechanism for the dominant negative effect of these mutations remains elusive, but converging evidence suggests that it may be caused by MC1R wild-type heterodimerization with variant receptors, which results in an allele specific dominant-negative effect on wild-type cell surface expression and the ensuing ability to stimulate cAMP production^42^. Indeed, patients 1 and 2 in this study were heterozygous for D84E and R151C but still had congenital RHC, freckles and fair skin (Fitzpatrick skin type 1), denoting a functional defect of MC1R. This heterozygote effect was further verified by the impairment of NDP-MSH-induced cAMP responses in PBMCs. In addition to the cAMP pathway, another major signaling cascade downstream MC1R is the extracellular signal-regulated kinase (ERK) pathway, which is triggered in a biased mode by some mutant MC1R, thus resulting in no loss of signaling activities^53^. However, the MC1R-associated ERK pathway is unlikely responsible for the beneficial effect of melanocortin therapy in our study, because the ERK pathway is known to exert a detrimental effect in podocytopathy^54^,^55^,^56^,^57^. Moreover, the compensation of other MCR for the loss of MC1R function was also unlikely to occur and contribute, because phenotypes of MC1R deficiency, including congenital RHC in patients and yellow coat color in mice, were not diminished but evidently observed.

If MC1R is unessential, what is the exact mechanism by which melanocortins protect the kidney from podocyte injury and proteinuria? Kidney is a quintessential effector organ of the melanocortin hormone system and the melanocortins might target the glomerulus directly^28^. However, which type of MCR is expressed in the kidney and in glomeruli remains highly controversial. For instance, MC3R, MC4R and MC5R were found in both cortex and medulla of murine kidneys^32^; whereas, Lee *et al*.^33^ only demonstrated MC1R and MC3R expression in rat kidney. Our group recently found that MC1R is expressed abundantly and predominantly by renal tubules but weakly by glomeruli, whereas MC5R is expressed weakly and sporadically by kidney interstitial cells but strongly by glomerular cells in rodents *in vivo* and *in vitro*^31^. Moreover, strong expression of MC5R and weak expression of MC2R have been demonstrated in human kidneys by examining human kidney specific complementary DNA^30^. To a certain extent, this is in agreement with the observation in sea bass where MC5R was abundantly expressed in the anterior and posterior kidneys. In stark contrast, Lindskog *et al*.^28^, identified MC1R as the dominant MCR in virtually all types of kidney parenchymal cells in humans and rats, including podocytes, glomerular endothelial cells, mesangial cells, and tubular epithelial cells. However, in a following study, selective MC1R agonists barely induced any cAMP response in podocytes^58^. The doubt on podocyte expression of MC1R as well as MC1R-mediated podocyte protection was further accentuated by the contradictory effects of MC1R agonists on proteinuria and podocyte injury observed in different animal models of glomerulopathies^59^. In aggregate, the type of MCR expressed in glomeruli still remains uncertain and warrants further validation. Nevertheless, even if MC1R is indeed expressed by podocytes in the kidney, this does not necessarily establish its role in podocyte protection. In support of this, evidence from other MC1R-expressing tissues, such as the synovial joint^60^,^61^, suggests that abundant MC1R expression is not sufficient for the arthritis**-**resolving effect of melanocortin treatment^62^.

In addition to autonomous podocyte injury, systemic pathogenic components are also implicated in the development of podocyte injury and proteinuria^63^. Most nephrotic glomerular diseases, including iMN and podocytopathies, like MCD and FSGS, are known to involve extrarenal pathogenic culprits targeting glomerular podocytes, such as podocyte reactive autoantibodies and circulating permeability factors, which are presumably produced by immune competent cells^64^,^65^. Melanocortins are known to possess a potent immune modulatory activity in most immune competence cells *via* diverse MCR, like MC3R and MC5R^8^. Therefore, a systemic effect might also contribute, at least in part, to the protection of melanocortin treatment observed in this study, although our *in vitro* data suggest a direct podocyte protective activity. However, due to the limitations of systemic administration of NDP-MSH and global MC1R deficiency in mice, this study is unable to determine if the MC1R-independent beneficial effect of melanocortin therapy in glomerular disease is mainly attributable to a podocyte autonomous action or immune regulation or both. Alternatively, non-receptor-mediated^34^ or non-canonical^66^ pathways downstream of melanocortins have been described and may also be possibly responsible for the beneficial effect of melanocortin therapy. Another limitation of this study is that the effect of melanocortin therapy was only tested in murine models of the short-term LPS-induced podocytopathy. Whether the MC1R-independent beneficial effect of melanocortin therapy could be generalized to other forms of long-term experimental glomerulopathy, such as Adriamycin nephropathy, is unknown and warrants further investigation.

In summary, ACTH therapy successfully induced remission of proteinuria in MC1R mutant red-haired patients with steroid resistant nephrotic syndrome. In mice with LPS-elicited podocytopathy, the melanocortin peptide NDP-MSH conferred a proteinuria reducing and podocyte protective effect that was completely preserved in MC1R null mice and podocytes. Our findings suggest that MC1R is not required for the proteinuria reducing and renoprotective effect of the melanocortin therapy in proteinuric glomerulopathies.

## Methods

### Clinical Data

We retrospectively evaluated patients with nephrotic syndrome who were treated with synthetic ACTH (Synacthen Depot, Swedish Orphan Biovitrum AB, Stockholm, Sweden) between March 2009 and October 2015 at the University Hospital of Lund in Sweden according to the protocols developed by Berg *et al*.^16^,^21^. Four patients with congenital RHC were identified. They all demonstrated clinical evidence of nephrotic syndrome. Two were diagnosed with iMN and the other 2 with primary FSGS as proven by kidney biopsy. After unsuccessful treatments with different immunosuppressive regimens, ACTH treatment was started and lasted for varying periods of time. Supportive adjuvant treatments were given to patients as needed, including angiotensin II receptor blockades and/or angiotensin converting enzyme inhibitors, statins, and diuretics. Clinical parameters such as serum albumin, serum creatinine and proteinuria were followed during the treatment period and after treatment withdrawal. Discarded or excess blood specimens were collected. DNA samples obtained from a healthy volunteer without RHC served as control. Complete remission was defined as stable or improved renal function (estimated glomerular filtration rate, eGFR, based on serum creatinine) with urinary protein to creatinine ratio (uACR) <50 mg/mmol (For uACR, the International System of Unit or SI is mg/mmol and the metric unit is mg/g^67^ with the conversion factor being 10); partial remission as stable or improved renal function with uACR <350 mg/mmol) and a 50% or greater reduction from peak values^68^. Failure to meet the above criteria was classified as no response. Given the small number of patients and the observational nature of this clinical study, the data are presented descriptively, and no formal statistical analyses were performed. The clinical part of this study conformed to the ethical guidelines of the 1975 Declaration of Helsinki and was approved by the Institutional Review Board of the Lund University Hospital. Written informed consent was obtained from each subject.

### Genomic DNA extraction and Sanger sequencing

Genomic DNA was extracted using the DNeasy Blood and Tissue kit (Qiagen, Valencia, CA, USA). The protein-coding region of MC1R gene was sequenced by Sanger method for DNA sequencing (Genewiz, Inc, South Plainfield, NJ, USA). The primers used for Sanger sequencing are as follows: forward: 5′-CAACGACTCCTTCCTGCTTC-3′, reverse: 5′-TCACACAGGAACCAGACCAC-3′.

### Isolation and treatment of PBMCs and cAMP assay

Blood samples were collected from the 4 red-haired patients and 1 control individual with wild-type MC1R. PBMCs were prepared by Ficoll-Paque density gradient centrifugation as previously elaborated^69^. PBMCs were seeded in 96-well plates at the density of 10^−4^cells/well, pretreated with 100 μM 3-isobutyl-1-methylxanthine (IBMX, Sigma-Aldrich, St. Louis, MO, USA), and stimulated with 10 μM forskolin (Sigma-Aldrich, St. Louis, MO, USA) or 10^−7^ M NDP-MSH^70^ (GL Biochem Ltd., Boston, MA, USA). Intercellular cAMP levels were determined by using the Amersham cAMP Biotrak Enzymeimmunoassay (EIA) System (GE Healthcare Life Sciences, Pittsburgh, PA, USA) according to the manufacturer’s introduction. Vehicle-induced cAMP formation in PBMCs derived from the wild-type individual was defined as 100%.

### Animal study

The Animal Care and Use Committees at Zhengzhou University and Rhode Island Hospital approved animal studies and they conformed to the United States Department of Agriculture regulations and the National Institutes of Health guidelines for humane care and use of laboratory animals.

### Generation of mice null for MC1R

*Mc1r*^E/e^ mice on a C57BL/6 genetic background were purchased from the Jackson Laboratory (Bar Harbor, ME, USA) and bred to generate the recessive yellow *Mc1r*^e/e^ mice null for MC1R^37^,^38^ and the wild-type (WT) control littermates. Blood and spot urine were collected from male Mc1r^e/e^ and WT mice at indicated ages and body weight measured. Mice were euthanized at indicated ages and kidneys excised, weighted and processed for histologic examination.

### Murine model of LPS-induced proteinuria and podocytopathy

Male mice aged 8 weeks were randomly assigned to the following treatments. A single dose of LPS (serotype: E. coli 0111: B4, Sigma-Aldrich, St. Louis, MO, USA) 200 μg or an equal volume of saline was given via intraperitoneal injection. NDP-MSH (0.6 μmol/kg *wt*, GL Biochem Ltd.)^71^ or saline was given *via* subcutaneous injection at 1 hour before and 12 hours after LPS injection. Mice were followed for 24 hours after LPS injection and euthanized. Six mice were randomly assigned to each group. Spot urine was collected before LPS injection and 12 and 24 hours after LPS injection.

### Urine Analyses

To discern the protein compositions in urine, equal amounts of urine samples were subjected to SDS-PAGE followed by Coomassie blue staining. Urine albumin concentration was measured using a mouse albumin enzyme-linked immunosorbent assay (ELISA) quantitation kit (Bethyl Laboratories Inc., Montgomery, TX). Urine creatinine concentration was measured by a creatinine assay kit (BioAssay Systems, Hayward, CA).

### Histologic studies and transmission electron microscopy

Formalin fixed kidneys were embedded in paraffin and prepared sections (3 μm thick). For general histologic analysis, sections were processed for Periodic acid-Schiff (PAS) staining. The kidney cortical specimens were cut into small pieces (1 mm^3^), fixed with 2.5% glutaraldehyde in phosphate buffered saline, pH 7.4, and embedded in Epon 812 (Polysciences, Warrington, PA, USA). Transmission electron micrographs were obtained using a Zeiss EM-10 electron microscope operated at 80 kV with absolute magnifications of 8000 or 20000. For examination of podocyte foot processes, 6 random electron microscopic fields of glomeruli per animal were examined. The morphologic features were assessed by a single observer in a blinded manner.

### Glomerular isolation

Mice were anesthetized and kidney perfused with 5 ml PBS containing 8 × 10^7^ Dynabeads M-450 (Dynal Biotech ASA, Oslo, Norway). Kidneys were immediately removed and placed in cold PBS. The renal capsule was removed, and the kidney sagittally cut into two halves. The medulla was dissected and discarded from each half. The remaining cortical tissue was minced into 1 mm^3^ pieces and digested in collagenase A (1 mg/ml) at 37 °C for 30 min with gently shaking. The tissue was pressed gently through a 100 μm cell strainer (BD Falcon, Bedford, MA, USA). The glomeruli containing Dynabeads were then gathered using a magnetic particle concentrator^72^. The isolated glomeruli were washed 3 times with cold PBS and then cultured in RPMI 1640 (Life Technologies, Grand Island, NY, USA) supplemented with 10% fetal bovine serum in incubator with 5% CO_2_ for subsequent studies.

### Cell culture and treatments

The enriched glomeruli were plated on collagen type I-coated dishes at 37 °C in RPMI 1640 supplemented with 10% Fetal Bovine Serum (FBS) in a humidified incubator with 5% CO_2_. Subculture of primary podocytes was performed by detaching the glomerular cells with 0.25% trypsin-EDTA (Invitrogen, Carlsbad, CA, USA), followed by sieving through a 40-μm cell strainer, and culture on collagen type I-coated dishes. Podocytes of passages 1 or 2 were characterized by the expression of multiple podocyte specific markers and used in all experiments^73^. Primary cultured podocytes were pretreated with 10^−7^ M NDP-MSH or vehicle for 30 min and then stimulated with 20 μg/ml LPS or saline for 24 h.

### Cell migration assay

Confluent monolayers of primary podocytes were scraped with a 10 μl pipette after different treatments and visualized at 24 h using an inverted microscope. Phase contrast micrographs were obtained at 0 h and 24 h after scratching. Data were calculated and expressed as the percent area closure, which represents the percent difference in wound area after 24 hours and were analyzed using Image J version 32 (NIH, Bethesda, MD) image processing program^50^.

### Albumin influx assay

A paracellular permeability influx assay was used to evaluate the filtration barrier function of podocyte monolayers^74^,^75^. Primarily cultured podocytes were seeded into the collagen coated transwell filters (3 μm pore, Corning, NY, USA) in the top chamber. After different treatments for 24 h, podocytes were washed twice with PBS supplemented with 1 mmol/L MgCl_2_ and CaCl_2_. The top chambers were then refilled with 0.15 ml RPMI 1640 and the bottom chambers were refilled with 1 ml RPMI 1640 supplemented with 40 mg/ml of Bovine Serum Albumin (BSA) and then incubated at 37 °C. At 1 h and 3 h, 20 μl medium in the top chambers were collected and prepared for albumin concentration assay by using a bicinchoninic acid protein assay kit (GenDEPOT, Barker, TX, USA).

### Western immunoblot

Cultured cells were lysed and kidney cortical specimens were homogenized in Radioimmunoprecipitation assay (RIPA) buffer supplemented with protease inhibitors. Samples were subjected to immunoblot analysis as previously described^76^. The antibodies against synaptopodin, podocin, p-GSK3β and glyceraldehyde-3-phosphate dehydrogenase (GAPDH) were purchased from Santa Cruz Biotechnology (Santa Cruz, CA, USA), those against B7-1 from R&D Systems (Minneapolis, MN, USA), and that against cleaved caspase-3 from Cell Signaling Technology (Danvers, MA, USA).

### Immunofluorescent staining and TUNEL staining

Cultured cells or frozen cryostat sections were fixed with 4% paraformaldehyde, permeabilized and stained with primary antibodies and followed by the Alexa Fluor-conjugated secondary antibodies (Life Technologies, Grand Island, NY, USA). Finally, cells were counterstained with 4′,6-diamidino-2-phenylindole (DAPI), mounted with Vectashield mounting medium (Vector Laboratories, Burlingame, CA, USA). As a negative control, the primary antibodies were replaced by preimmune IgG from the same species. Apoptotic cell death in cell cultures or kidney sections was detected by using the TUNEL kit (Roche Molecular Biochemicals, Mannheim, Germany) as described previously^31^. Each staining was visualized using a fluorescence microscope with identical light exposure settings between different groups.

### Statistical analyses

Statistical analyses were performed by SPSS 22 (IBM Corporation, Armonk, New York, USA). All data are expressed as mean ± SD. Unless otherwise indicated, all experimental observations were repeated six times. Statistical analysis of the data over time from multiple groups was performed by repeated measures ANOVA followed by Fisher’s Least Significant Difference (LSD) tests. Data from two groups were compared by Student’s t-test. *P* < 0.05 was considered significant.

## Additional Information

**How to cite this article**: Qiao, Y. *et al*. MC1R is dispensable for the proteinuria reducing and glomerular protective effect of melanocortin therapy. *Sci. Rep.* **6**, 27589; doi: 10.1038/srep27589 (2016).

## Figures and Tables

**Table 1:** Baseline data for nephrotic patients with congenital red hair color, and duration and outcomes of ACTH therapy.

Patient	Gender	Age (y)	Renal biopsy	Previous Immunosuppression	Steroid response	Duration of ACTH therapy (months)	ACEI/ARB	uACR (mg/mmol)		eGFR (ml/min/1.73 m^2^)		Outcome	Adverse effects	MC1R mutations
								Before	After	Before	After			
1	Male	65	iMN (II)	Steriods	Steroid resistance	7	Yes	485	0.85	54	56	Complete remission	No	D84E
2	Female	45	iMN (II~III)	Steriods + Tacrolimus	Steroid resistance	21	Yes	244	7.5	92	113	Complete remission	No	R151C
3	Male	18	FSGS	Steriods + MMF, Cyclosporine, Tacrolimus	Steroid resistance	>97	Yes	983	1.9	<20	63	Complete remission	Pulmonary aspergillosis	R151C
4	Male	53	FSGS	Steriods + Cyclosporine, Tacrolimus	Steroid resistance	52	Yes	300	1.4	59	72	Complete remission	No	R151C

Abbreviations: ACEI, angiotensin-converting enzyme inhibitors; ARB, angiotensin II receptor blockers; eGFR, estimated glomerular filtration rate; FSGS, Focal segmental glomerulosclerosis; iMN, idiopathic membranous nephropathy; MMF, mycophenolate mofetil; uACR, urinary albumin to creatinine ratio.
