# Author Correction: MC1R is dispensable for the proteinuria reducing and glomerular protective effect of melanocortin therapy

**DOI:** 10.1038/s41598-020-72744-7

**Published:** 2020-09-21

**Authors:** Yingjin Qiao, Anna-Lena Berg, Pei Wang, Yan Ge, Songxia Quan, Sijie Zhou, Hai Wang, Zhangsuo Liu, Rujun Gong

**Affiliations:** 1 Institute of Nephrology, Blood Purification Center, the First Affiliated Hospital of Zhengzhou University, Zhengzhou, China; 2 Division of Kidney Disease and Hypertension, Department of Medicine, Rhode Island Hospital, Brown University School of Medicine, Providence, Rhode Island, USA; 3 Department of Nephrology, Lund University Hospital, Lund, Sweden; 4 Department of Pathology, Rhode Island Hospital, Brown University School of Medicine, Providence, Rhode Island, USA

Correction to: *Scientific Reports*, 10.1038/srep27589, Published online 08 June 2016

This Article contains errors.

In Figure 8a, at time 0 h, the images for Control e−/e−, LPS WT, and LPS e−/e−, were inadvertently taken from the Control WT condition. The correct Figure 8a is now included below as Figure 1.

**Figure 1:**
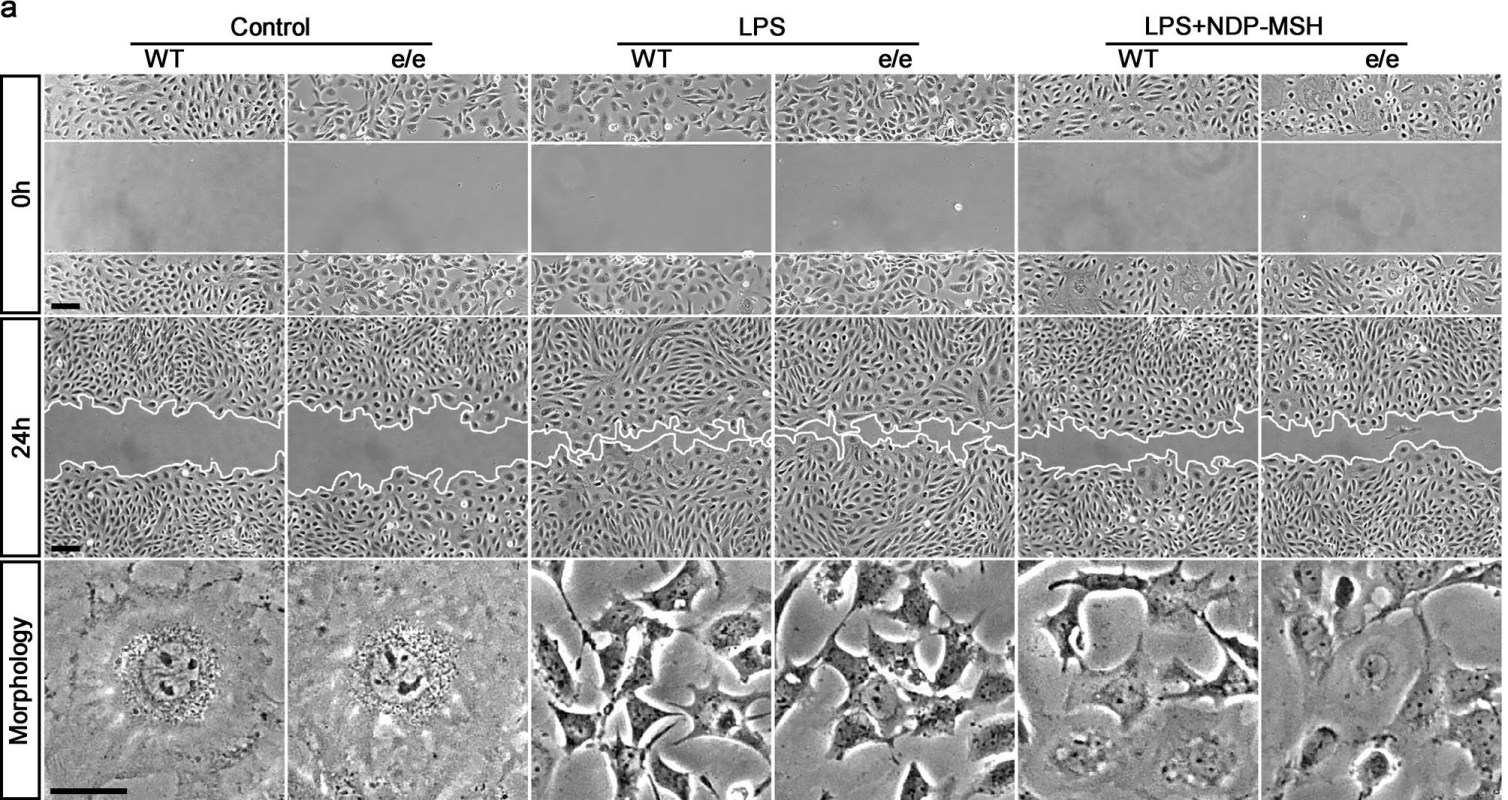
(**a**) Phase-contrast micrographs were taken immediately after wounding (0 h) and after migration for 24 h. Scale bar = 100 μm. Cell morphology at 24 h was taken under high-power fields. LPS injury resulted in marked podocyte shrinkage and this effect was abrogated by NDP-MSH. Scale bar = 20 μm.

The errors do not affect the results or conclusions of the study.

